# Revolutionizing hematological disorder diagnosis: unraveling the role of artificial intelligence

**DOI:** 10.1097/MS9.0000000000003227

**Published:** 2025-04-02

**Authors:** Emmanuel Ifeanyi Obeagu

**Affiliations:** Department of Biomedical and Laboratory Science, Africa University, Mutare, Zimbabwe

**Keywords:** artificial intelligence, big data, diagnostics, hematological disorders, machine learning

## Abstract

The integration of artificial intelligence (AI) into medical diagnostics is transforming the landscape of healthcare, particularly in hematology. AI technologies, leveraging advanced machine learning algorithms and big data analytics, are revolutionizing the diagnosis of hematological disorders such as anemia, leukemia, and lymphoma. This review explores how AI enhances diagnostic accuracy, efficiency, and patient outcomes by processing complex datasets and identifying patterns beyond human capability. AI-driven advancements in hematology include innovations in image analysis, genomic data interpretation, and predictive modeling. Convolutional neural networks analyze blood smear images with high precision, detecting subtle morphological abnormalities and classifying blood cells. Machine learning models interpret genomic data, identifying genetic mutations linked to specific disorders, which is crucial for diagnosing hereditary blood conditions and cancers. Predictive modeling, based on historical patient data, forecasts disease progression and treatment responses, enabling personalized patient management. Despite the promising benefits, the implementation of AI in hematological diagnostics faces challenges such as ensuring data quality and integration, addressing ethical and regulatory concerns, and maintaining transparency and accountability of AI algorithms. Ongoing research and collaboration between clinicians, data scientists, and regulatory bodies are essential to advance AI capabilities and ensure safe and effective solutions. As AI continues to evolve, its integration into hematology holds significant promise for improving diagnostic practices and patient care.

## Introduction

The field of hematology, which focuses on the study of blood, blood-forming organs, and blood diseases, has witnessed significant advancements over the past few decades^[1]^. Despite these advancements, diagnosing hematological disorders remains a complex and challenging task. Traditional diagnostic methods, which rely heavily on manual examination and interpretation, are often time-consuming and subject to human error. With the emergence of artificial intelligence (AI), a new frontier has opened up, offering promising solutions to these challenges and revolutionizing the way hematological disorders are diagnosed. AI, particularly machine learning, has shown great potential in transforming various sectors, including healthcare^[2]^. Machine learning algorithms can analyze vast amounts of data, recognize patterns, and make predictions with high accuracy. In hematology, these capabilities translate into more precise and efficient diagnostic processes. By leveraging AI, clinicians can now access tools that assist in the rapid and accurate diagnosis of a wide range of blood disorders, from common conditions like anemia to more complex diseases like leukemia and lymphoma. One of the most significant contributions of AI to hematology is in the area of image analysis. The examination of blood smears, a critical component in diagnosing many hematological disorders, has traditionally relied on the expertise of trained pathologists. However, AI-powered image analysis systems, such as convolutional neural networks (CNNs), have demonstrated the ability to analyze microscopic images with a level of precision that rivals human experts^[3]^. These systems can detect and classify different types of blood cells, identify morphological abnormalities, and even predict the presence of certain disorders, all in a fraction of the time required by traditional methods.

**HIGHLIGHTS**

- AI enhances hematological diagnosis: AI technologies improve diagnostic accuracy for hematological disorders.
- Personalized treatment strategies: AI facilitates personalized medicine in hematology.
- Increased Efficiency and Speed: AI streamlines diagnostic processes and treatment workflows in hematology, resulting in faster decision-making, reduced time for analysis, and more efficient patient care.
- Cost-Effectiveness of AI Tools: AI applications in hematology offer cost-effective solutions.
- Future Research and Development: AI presents future research opportunities in hematology.

In addition to image analysis, AI has also made strides in the interpretation of genomic data^[4]^. The genetic basis of many hematological disorders necessitates the analysis of complex genomic sequences to identify mutations and variations that contribute to disease. AI algorithms can process and interpret these large datasets more efficiently than human researchers, providing valuable insights that are crucial for accurate diagnosis^[3]^. This capability is especially important in diagnosing hereditary blood disorders and cancers, where genetic information plays a pivotal role in understanding the disease. Predictive modeling is another area where AI is making a significant impact. By analyzing historical patient data, machine learning models can identify patterns that predict disease progression and treatment outcomes. This predictive power enables personalized medicine, where treatment plans are tailored to the individual characteristics of each patient, improving overall outcomes. For instance, in conditions like leukemia, AI can help predict which patients are more likely to respond to specific therapies, thus optimizing treatment strategies. Despite the clear advantages, the integration of AI into hematological diagnostics is not without challenges. One of the primary concerns is the quality and integration of data from diverse sources. Ensuring that data is accurate, standardized, and interoperable is essential for the effective use of AI. Moreover, the complexity of AI algorithms often makes them difficult to interpret, raising questions about transparency and accountability. Clinicians must understand how these algorithms arrive at their conclusions to trust and effectively use AI-driven diagnostic tools. Ethical and regulatory considerations also play a crucial role in the adoption of AI in healthcare. Issues such as data privacy, algorithmic bias, and the potential for over-reliance on AI systems need to be carefully addressed. Developing robust frameworks to govern the use of AI in diagnostics is essential to protect patient rights and ensure that AI applications are used responsibly and ethically.

## Aim

The aim of this review article is to explore and elucidate the transformative impact of AI on the diagnosis and management of hematological disorders.

## Rationale

The rationale for this review article stems from the significant and evolving role of AI in the field of hematology. As AI technologies advance, they offer new opportunities and present challenges that can profoundly impact the diagnosis, treatment, and management of hematological disorders. AI technologies are rapidly evolving and have demonstrated transformative potential in various medical fields, including hematology. The introduction of advanced machine learning algorithms, predictive analytics, and automated diagnostic tools has created new possibilities for improving patient care. Despite these advancements, there remains a need for a comprehensive review to systematically evaluate the current state of AI technologies in hematology, their applications, and their potential to reshape clinical practices. This review aims to bridge this gap by summarizing the latest developments and assessing their implications for the field. There is a growing interest and substantial investment in AI technologies for healthcare, including hematology. Research funding, clinical trials, and industry partnerships are increasingly focused on exploring AI’s potential to enhance diagnostic accuracy, develop personalized treatment plans, and improve patient outcomes^[3,4]^. The rationale for this review is to provide a detailed examination of how these investments are translating into real-world applications and to highlight the ways in which AI is advancing the practice of hematology. This review aims to inform stakeholders, including researchers, clinicians, and policymakers, about the progress and future directions of AI in this domain.

While there is a wealth of literature on AI in various medical disciplines, there is a need for a comprehensive review that specifically addresses the applications of AI in hematology. Existing reviews may focus on general AI applications or specific technologies without providing a holistic view of their impact on hematological disorders. This review seeks to fill this gap by offering a detailed and focused exploration of AI’s role in hematology, including diagnostic innovations, treatment advancements, and challenges. By providing an in-depth overview, the review aims to serve as a valuable resource for understanding the current landscape of AI in hematology. The integration of AI into clinical practice presents both opportunities and challenges. While AI technologies have the potential to enhance diagnostic capabilities, optimize treatment strategies, and streamline healthcare processes, there are also significant challenges related to data quality, algorithmic biases, and ethical concerns. This review aims to address both the benefits and challenges of AI in hematology, providing a balanced perspective on how these technologies are being implemented and the hurdles that must be overcome. By exploring these aspects, the review seeks to guide future research and practice in AI-driven hematology. AI in hematology is a rapidly evolving field with numerous opportunities for future research and development. There is a need to identify emerging trends, potential research areas, and innovative applications that could shape the future of hematological care. This review aims to outline future directions for AI research in hematology, including new technologies, methodologies, and potential breakthroughs. By highlighting these opportunities, the review seeks to inspire new research initiatives and drive forward the development of AI applications in hematology.

Case studies demonstrating the impact of AI technologies on patient outcomes provide valuable evidence of their effectiveness and potential. However, there is often a lack of comprehensive reviews that synthesize these case studies and extract key lessons for clinical practice. This review aims to present and analyze case studies that showcase the real-world benefits of AI applications in hematology, offering insights into how these technologies have improved diagnostic accuracy, treatment outcomes, and patient care. Effective implementation of AI technologies in clinical settings requires a clear understanding of their benefits, limitations, and practical applications. This review provides an overview of how AI technologies are being used in clinical practice and offers guidance on how to successfully integrate these tools into routine care. By providing practical insights and best practices, the review aims to support clinicians and healthcare providers in adopting AI technologies to enhance patient care. The deployment of AI technologies in healthcare raises important ethical and regulatory questions. Issues such as data privacy, algorithmic transparency, and the potential for bias in AI systems are critical considerations for the responsible development and use of AI in hematology. This review aims to address these ethical and regulatory issues, promoting awareness and encouraging thoughtful discussions about the responsible implementation of AI technologies in clinical practice. There is a need for ongoing education and knowledge dissemination about the role of AI in hematology. Clinicians, researchers, and students require up-to-date information about the latest advancements and applications of AI technologies. This review seeks to enhance knowledge dissemination by providing a comprehensive and accessible resource on AI in hematology, aimed at educating and informing the broader medical community. The development and implementation of AI technologies in hematology benefit from collaboration between researchers, clinicians, and industry professionals. This review aims to facilitate such collaboration by providing a detailed overview of current AI applications, highlighting successful case studies, and identifying future research opportunities. By fostering dialogue and collaboration, the review seeks to advance the collective efforts of the hematology community in improving patient care through AI technologies.

## Understanding AI in hematology

Understanding the role of AI in hematology involves grasping both the principles of AI and the unique applications within the domain of blood disorders. ML algorithms enable computers to learn and improve from experience without explicit programming^[5]^. In hematology, ML algorithms are trained on large datasets to recognize patterns and make predictions. DL, a subset of ML, involves neural networks with multiple layers that mimic the human brain’s structure. Deep neural networks are used in complex pattern recognition tasks in hematology, like image analysis. NLP allows computers to understand, interpret, and generate human language. In hematology, NLP is applied to analyze and extract insights from medical texts and research papers. AI assists in analyzing blood smears, bone marrow aspirates, and histopathological slides. It helps identify and categorize blood cells, aiding in diagnosing conditions like anemias, leukemias, and lymphomas. AI algorithms assist hematologists by suggesting potential diagnoses based on patient data and medical literature^[6]^. These systems help in considering a wide range of possibilities for complex cases. AI models analyze patient data to predict disease progression, treatment responses, and potential complications. They assist in identifying high-risk patients and planning appropriate interventions. AI helps in tailoring treatment plans based on individual patient profiles, including genetic markers and treatment response patterns. This personalized approach aims for more effective and targeted therapies. AI aids in sifting through vast amounts of medical literature, assisting researchers in identifying relevant studies, extracting valuable insights, and accelerating the pace of discovery in hematology.

## The role of AI in hematology

AI is increasingly transforming various medical fields, and hematology is no exception. The application of AI in hematology promises to enhance diagnostic accuracy, streamline workflows, and improve patient outcomes. This section delves into the key roles AI plays in hematology, focusing on machine learning algorithms, big data analytics, image analysis, genomic data interpretation, and predictive modeling.

## Machine learning algorithms

Machine learning, a subset of AI, involves training algorithms to recognize patterns and make predictions based on data. In hematology, machine learning models analyze complex datasets such as blood smear images, genetic profiles, and patient histories^[7]^. These models can identify subtle abnormalities that may be missed by human observers, thereby improving diagnostic accuracy and reducing the likelihood of errors. Algorithms such as support vector machines, random forests, and neural networks are commonly used to develop predictive models for diagnosing blood disorders.

## Big data analytics

The vast amount of data generated in healthcare, including electronic health records (EHRs), laboratory results, and clinical studies, presents both a challenge and an opportunity for hematology. AI excels in processing and analyzing big data, extracting valuable insights that can enhance diagnostic precision^[8]^. By integrating data from various sources, AI systems can provide comprehensive diagnostic assessments, identify trends, and uncover correlations that may not be apparent through traditional analysis methods. This holistic approach can lead to more informed decision-making and better patient outcomes.

## Image analysis

AI-powered image analysis has revolutionized the examination of blood smears and bone marrow biopsies. CNNs, a type of deep learning algorithm, can analyze microscopic images with high accuracy^[9]^. These systems can detect and classify different types of blood cells, identify morphological abnormalities, and predict the presence of specific hematological disorders. For example, AI algorithms can distinguish between different types of anemia, detect early signs of leukemia, and assess the severity of conditions like sickle cell disease. This capability not only enhances diagnostic accuracy but also speeds up the diagnostic process.

## Genomic data interpretation

Genomic data plays a crucial role in diagnosing and understanding hematological disorders. AI algorithms can analyze genetic sequences to identify mutations and variations associated with specific conditions^[8]^. This capability is particularly valuable in diagnosing hereditary blood disorders and cancers, where genetic factors are critical. For instance, AI can help identify genetic markers for predisposition to leukemia or lymphoma, enabling early intervention and personalized treatment plans. Moreover, AI-driven genomic analysis can assist in understanding the molecular mechanisms underlying these disorders, leading to the development of targeted therapies.

## Predictive modeling

Predictive modeling is another area where AI is making a significant impact in hematology^[10]^. By analyzing historical patient data, machine learning models can identify patterns and predict disease progression and treatment outcomes. This predictive power enables personalized medicine, where treatment plans are tailored to the individual characteristics of each patient. For instance, in conditions like leukemia, AI can help predict which patients are more likely to respond to specific therapies, optimize treatment strategies, and improve overall outcomes. Predictive models can also aid in risk stratification, helping clinicians identify high-risk patients who may benefit from more aggressive treatment or closer monitoring.

## Integration into clinical practice

Integrating AI into clinical practice requires overcoming several challenges, including ensuring data quality, standardization, and interoperability^[11]^. High-quality, standardized data is essential for training accurate and reliable AI models. Additionally, AI systems must be interoperable with existing healthcare infrastructure, such as EHR systems, to facilitate seamless data exchange and integration. Collaboration between clinicians, data scientists, and healthcare administrators is crucial to address these challenges and ensure the successful implementation of AI technologies in hematology.

## Applications of AI in hematological disorder diagnosis

AI has diverse applications in the diagnosis of hematological disorders, revolutionizing traditional approaches and enhancing diagnostic accuracy^[12]^. AI algorithms assist in the identification and classification of various blood cells in microscopic images, aiding in diagnosing conditions like anemias, leukemias, and lymphomas. AI helps in analyzing bone marrow aspirates or biopsies, facilitating the identification of abnormal cell populations indicative of hematologic malignancies or other disorders. AI-based systems aid hematologists by suggesting potential diagnoses based on patient data, laboratory results, and medical literature, providing comprehensive differential diagnoses for complex cases. AI-driven models help in recommending appropriate treatment options by analyzing patient-specific data, considering disease characteristics, treatment responses, and potential complications^[13]^.

AI tools predict disease progression, recurrence risk, and outcomes for patients with hematological disorders based on individual patient profiles, aiding in risk stratification and personalized treatment planning^[13]^. AI assists in predicting response to therapy, guiding hematologists in determining prognosis and planning follow-up strategies for better patient management. AI supports genomic analysis, identifying genetic mutations or aberrations associated with specific hematological disorders. This aids in tailoring treatments based on individual genetic profiles, facilitating personalized medicine approaches. AI models analyze genetic data to predict patient responses to specific drugs or therapies, optimizing treatment selection and minimizing adverse effects. AI algorithms sift through vast amounts of medical literature, extracting relevant information, and identifying emerging trends or insights in hematology, aiding researchers and clinicians in staying updated with the latest advancements. AI-powered telehematology platforms facilitate remote consultations and second opinions, enabling patients in underserved areas to access expert opinions and specialized care. These applications demonstrate how AI contributes to enhancing the accuracy, efficiency, and personalized nature of hematological disorder diagnosis. By leveraging AI’s capabilities in data analysis, pattern recognition, and predictive modeling, hematologists can make more informed decisions, leading to improved patient outcomes and more targeted treatments^[14]^.

## Advantages of AI in hematological diagnosis

The integration of AI into hematological diagnostics is transforming the field by providing significant advantages over traditional methods. These advantages include increased diagnostic accuracy, efficiency, cost-effectiveness, personalized treatment plans, and enhanced data analysis capabilities. This section explores the various benefits that AI brings to hematological diagnosis.

## Increased diagnostic accuracy

The integration of AI in hematological diagnostics significantly enhances diagnostic accuracy, providing more reliable and precise results compared to traditional methods^[15]^. AI’s advanced capabilities in pattern recognition, consistency, and data processing play a crucial role in improving diagnostic outcomes for various blood disorders. CNNs are particularly effective in analyzing medical images, such as blood smears and bone marrow biopsies. These networks can identify intricate patterns and subtle morphological changes in blood cells that may indicate specific hematological disorders. For instance, CNNs can distinguish between different types of white blood cells and detect abnormal cell shapes or sizes associated with conditions like leukemia and anemia. AI algorithms can automate the detection of rare and abnormal cells in a large dataset, significantly reducing the time and effort required for manual examination. This automation not only speeds up the diagnostic process but also ensures that rare abnormalities, which could be easily missed by human eyes, are accurately identified. AI can analyze complex genomic sequences to identify mutations and genetic variations associated with hematological disorders. Machine learning models are trained to recognize specific genetic markers linked to diseases such as sickle cell anemia, thalassemia, and various forms of leukemia. This precise identification is crucial for diagnosing hereditary blood disorders and understanding their molecular basis. AI algorithms can integrate data from genomics, transcriptomics, proteomics, and metabolomics to provide a comprehensive diagnostic overview. This multi-omics approach enhances the accuracy of diagnosis by considering various biological layers and their interactions, leading to a more thorough understanding of the disease^[16]^.

Traditional diagnostic methods often rely on the subjective interpretation of visual data by pathologists, which can lead to variability in diagnoses. AI systems provide objective analysis based on predefined algorithms, ensuring consistent and repeatable results regardless of the individual performing the diagnosis^[14]^. AI algorithms follow standardized protocols for data analysis, reducing the risk of inconsistencies that may arise from human fatigue or cognitive biases. This standardization is particularly important in hematology, where the accurate classification of blood cell types and detection of abnormalities are critical for diagnosis. AI can process and analyze vast amounts of data more efficiently than human diagnosticians. In cases involving complex data, such as high-throughput genomic sequencing or comprehensive blood panel tests, AI systems can identify patterns and correlations that might be overlooked by humans. This capability is especially valuable in diagnosing rare or complex hematological disorders. AI systems can learn and improve over time by incorporating new data and feedback from diagnostic outcomes. This continuous learning process ensures that AI algorithms remain up-to-date with the latest medical knowledge and practices, further enhancing their diagnostic accuracy.

AI can detect early markers of hematological disorders before clinical symptoms become apparent. For example, AI algorithms can identify early genetic changes or subtle morphological abnormalities in blood cells that indicate the onset of diseases such as leukemia^[17]^. Early detection enables timely intervention, improving the prognosis and treatment outcomes for patients. AI systems can monitor changes in patient data over time, identifying trends and potential issues before they escalate. This proactive approach to patient management helps in the early diagnosis and treatment of chronic hematological conditions, reducing the risk of complications. AI can analyze individual patient data, considering genetic, clinical, and environmental factors to provide personalized diagnostic insights. This personalized approach ensures that diagnoses are tailored to the specific characteristics of each patient, enhancing the accuracy and relevance of the diagnosis. AI-driven diagnostics support precision medicine by identifying the most appropriate treatment options based on the patient’s unique genetic and clinical profile. This precision ensures that patients receive the most effective therapies, reducing the trial-and-error approach often associated with traditional treatment methods.

## Efficiency and speed

The integration of AI into hematological diagnostics has significantly enhanced the efficiency and speed of the diagnostic process^[18]^. AI-powered image analysis tools, particularly those using CNNs, can process and interpret blood smear images much faster than human pathologists. These tools can automatically identify and classify different types of blood cells, detect abnormalities, and generate diagnostic reports within minutes. This rapid analysis is crucial in clinical settings where timely diagnosis can significantly impact patient outcomes. Similarly, AI algorithms can quickly analyze bone marrow biopsy samples, identifying malignant cells and other abnormalities. This speed is particularly important in diagnosing and monitoring hematological malignancies such as leukemia and lymphoma. AI algorithms can process and analyze vast amounts of genomic data generated by high-throughput sequencing technologies. Machine learning models can quickly identify genetic mutations and variations associated with hematological disorders, providing insights into the molecular basis of diseases. This rapid interpretation is essential for diagnosing genetic blood disorders and developing personalized treatment plans. AI systems can integrate data from multiple omics layers, including genomics, transcriptomics, proteomics, and metabolomics, to provide a comprehensive diagnostic overview. This integration enables faster and more accurate identification of disease biomarkers and underlying mechanisms.

AI can automate routine tasks such as data entry, documentation, and initial data analysis. By reducing the time and effort required for these tasks, AI allows healthcare professionals to focus on more complex and critical aspects of patient care. This automation streamlines workflows and enhances overall efficiency in hematological diagnostics. AI systems can perform initial diagnostic assessments by analyzing patient data and generating preliminary reports. These assessments can serve as a valuable starting point for clinicians, providing them with a comprehensive overview of the patient’s condition and helping to prioritize further diagnostic tests and evaluations. AI can seamlessly integrate with EHR systems, facilitating the automatic extraction and analysis of patient data. This integration ensures that all relevant information is readily available for diagnostic assessments, reducing the time required for data retrieval and manual input. AI systems can enhance coordination between different departments and specialists involved in hematological care. By providing a unified platform for data sharing and communication, AI improves collaboration and ensures that all stakeholders have access to the latest diagnostic information^[18]^.

AI-powered wearable devices and sensors can continuously monitor patients’ vital signs and other health parameters. These devices can detect early signs of hematological complications, such as abnormal bleeding or clotting, and alert healthcare providers in real-time. This continuous monitoring enables timely interventions and reduces the risk of adverse events. AI facilitates remote monitoring of patients with chronic hematological conditions, allowing healthcare providers to track disease progression and treatment responses from a distance. This remote monitoring capability is particularly valuable for patients in remote or underserved areas, ensuring they receive timely and appropriate care. AI-enabled point-of-care testing devices can provide rapid diagnostic feedback at the patient’s bedside or in remote settings. These devices use AI algorithms to analyze blood samples and generate diagnostic results within minutes, enabling immediate clinical decision-making and reducing the need for time-consuming laboratory tests. AI systems can analyze patient data in real-time, providing instant diagnostic insights and recommendations. This real-time analysis is crucial in acute care settings, where quick decision-making can significantly impact patient outcomes^[16]^.

AI-powered clinical decision support systems (CDSS) can assist healthcare providers by offering evidence-based recommendations and highlighting potential diagnostic considerations. These systems analyze patient data, cross-reference it with medical knowledge databases, and provide clinicians with actionable insights to support their diagnostic decisions. AI algorithms can stratify patients based on their risk of developing complications or experiencing disease progression. This stratification helps clinicians prioritize high-risk patients and allocate resources more effectively, ensuring that patients receive the appropriate level of care. AI can automate the generation of diagnostic reports, reducing the time required for manual report writing and review. This automation ensures that diagnostic results are available to clinicians and patients more quickly, enabling timely initiation of treatment. AI accelerates research and development in hematology by rapidly analyzing large datasets and identifying new disease markers and therapeutic targets. This acceleration leads to faster development of diagnostic tools and treatments, ultimately benefiting patients through more advanced and effective care options^[14]^.

## Cost-effectiveness

The adoption of AI in hematological diagnostics is leading to significant cost savings and efficiency improvements across healthcare systems^[19]^. By automating labor-intensive tasks, reducing diagnostic errors, optimizing resource allocation, and enhancing patient management, AI contributes to the cost-effectiveness of hematological diagnostics. AI algorithms can automatically analyze blood smears, bone marrow biopsies, and other diagnostic images, reducing the need for manual examination by pathologists. This automation not only speeds up the diagnostic process but also lowers the labor costs associated with manual image analysis. AI systems can automate data entry, documentation, and initial data analysis, minimizing the time and effort required for these routine tasks. This reduction in manual labor allows healthcare professionals to focus on more complex and high-value activities, improving overall productivity. By handling routine and repetitive tasks, AI frees up skilled healthcare professionals to perform more specialized and critical duties. This efficient use of personnel ensures that valuable human resources are optimally allocated, enhancing the cost-effectiveness of diagnostic services. AI tools can assist in training and development by providing real-time feedback and guidance to junior healthcare professionals. This support reduces the need for extensive training programs and allows new staff to quickly become proficient in diagnostic procedures, further reducing costs^[20]^.

AI algorithms provide consistent and precise analysis, reducing the likelihood of diagnostic errors. Accurate diagnoses are essential for effective patient management, as misdiagnoses can lead to unnecessary treatments, additional tests, and prolonged hospital stays, all of which contribute to higher healthcare costs. AI can identify diseases at earlier stages by detecting subtle changes in diagnostic data that precede clinical symptoms. Early detection allows for timely intervention, reducing the costs associated with advanced disease treatment and improving patient outcomes. AI-driven diagnostic tools can streamline the diagnostic process by accurately identifying the necessary tests and procedures. This targeted approach reduces the need for redundant or unnecessary tests, saving both time and resources. By providing accurate initial diagnoses, AI minimizes the need for costly follow-up procedures and additional testing to confirm or correct earlier results. This reduction in follow-up procedures leads to significant cost savings for healthcare systems^[19]^.

AI can optimize the use of diagnostic equipment by scheduling tests and procedures based on patient needs and equipment availability. This optimization ensures that expensive diagnostic tools are used efficiently, reducing idle time and increasing throughput. AI systems can assist in managing inventory by predicting the demand for diagnostic supplies and reagents. Accurate inventory management prevents overstocking or shortages, reducing waste and ensuring that resources are available when needed. By enabling faster and more accurate diagnoses, AI can reduce the duration of hospital stays. Shorter hospital stays lower the costs associated with inpatient care, freeing up beds and resources for other patients. AI-driven diagnostic tools can facilitate efficient outpatient care by providing quick and accurate test results. This efficiency reduces the need for multiple visits and follow-up appointments, lowering the overall cost of care^[16]^. AI can analyze individual patient data to develop personalized treatment plans, ensuring that therapies are tailored to the specific characteristics and needs of each patient. Personalized treatment plans improve treatment efficacy, reduce the risk of adverse effects, and minimize the costs associated with trial-and-error approaches to therapy. AI-driven predictive models can forecast disease progression and treatment outcomes, allowing clinicians to proactively adjust treatment plans. This proactive management reduces the likelihood of complications and hospitalizations, leading to cost savings. AI can assist in the ongoing monitoring of patients with chronic hematological conditions, ensuring that any changes in their condition are promptly identified and addressed. Efficient chronic disease management reduces the need for emergency interventions and hospitalizations, lowering long-term healthcare costs. AI-powered applications can support patient adherence to treatment plans by providing reminders, educational resources, and real-time feedback. Improved adherence leads to better health outcomes and reduces the costs associated with non-compliance, such as disease relapse or progression^[21]^.

AI-driven diagnostic tools can be deployed in various settings, including remote and underserved areas, providing wider access to high-quality diagnostic services^[18]^. This scalability ensures that more patients receive timely and accurate diagnoses, reducing the overall burden on healthcare systems. By providing accurate and timely diagnoses, AI reduces the need for patients to undergo multiple tests and visits, lowering their out-of-pocket expenses. Additionally, early and effective treatment minimizes the indirect costs associated with lost productivity and long-term disability. AI helps healthcare systems optimize the use of resources, ensuring that diagnostic tools, personnel, and supplies are used efficiently. This optimization contributes to the sustainability of healthcare systems by reducing waste and improving cost-effectiveness. The cost savings achieved through AI-driven diagnostics can be reinvested in healthcare infrastructure, research, and development, further enhancing the quality and accessibility of healthcare services^[22]^.

## Personalized treatment plans

AI is transforming the field of hematology by enabling personalized treatment plans that are tailored to the unique needs of individual patients. AI algorithms can integrate diverse types of data, including genomic, transcriptomic, proteomic, and metabolomic information^[19]^. By analyzing these multi-omics datasets, AI can identify specific disease mechanisms, biomarkers, and therapeutic targets for each patient. For example, in the management of leukemia, AI can analyze genetic mutations, gene expression profiles, and protein markers to pinpoint the most effective treatment options tailored to the patient’s unique molecular profile. AI enhances the discovery and validation of biomarkers that are crucial for personalized treatment. Through machine learning models, researchers can uncover new biomarkers from large-scale data sets that might be missed using traditional methods. For instance, AI can identify specific genetic mutations or protein expressions associated with drug resistance in cancer patients, leading to the development of targeted therapies and personalized treatment plans. AI-driven predictive models use historical patient data to forecast how individuals will respond to specific treatments. By analyzing data from previous cases, these models can predict the effectiveness of different therapies for current patients. For instance, in managing chronic myeloid leukemia (CML), AI models can predict responses to tyrosine kinase inhibitors based on genetic mutations and treatment history, allowing clinicians to select the most effective drug regimen for each patient. AI tools can predict the future course of a disease by analyzing patient data trends over time. This predictive capability allows clinicians to anticipate disease progression and adjust treatment plans proactively. For example, in patients with myelodysplastic syndromes (MDS), AI algorithms can forecast the risk of progression to acute myeloid leukemia (AML) and help determine the timing for more aggressive treatments or interventions^[23]^.

AI-powered wearable devices and sensors can continuously monitor patient health metrics such as blood pressure, heart rate, and blood glucose levels^[20]^. These devices provide real-time data that can be analyzed to assess treatment efficacy and make timely adjustments. For example, wearables can monitor symptoms and side effects of treatment in patients with anemia, allowing for adjustments in therapy or supportive care as needed. AI facilitates remote monitoring of patients, allowing clinicians to track disease status and treatment responses without requiring frequent office visits. Through telemedicine platforms, AI can analyze patient-reported outcomes and electronic health record data to provide ongoing treatment recommendations and adjustments, enhancing patient management for conditions such as sickle cell disease. AI algorithms can support dynamic treatment adjustments by analyzing real-time patient data and predicting the outcomes of potential treatment changes. For instance, in the treatment of lymphoma, AI can analyze response data from ongoing treatment to suggest modifications in therapy regimens, dosage adjustments, or additional supportive treatments. AI can guide personalized follow-up care plans based on the patient’s treatment response and recovery progress. By evaluating patient data and treatment outcomes, AI can recommend specific follow-up tests, additional therapies, or lifestyle modifications to optimize long-term health outcomes^[24]^.

AI can analyze genetic variations that affect drug metabolism and efficacy through pharmacogenomic data. This analysis enables the selection of drugs that are best suited to an individual’s genetic profile, minimizing adverse effects and enhancing therapeutic effectiveness. For example, AI can identify patients who are likely to benefit from specific targeted therapies for hematological cancers based on their genetic profiles. AI models can predict potential drug resistance by analyzing genetic mutations and treatment history. This prediction helps in selecting alternative therapies or combination treatments that are more likely to overcome resistance. In cases of AML, AI can predict which patients are at higher risk for resistance to certain chemotherapies and guide the choice of alternative treatment strategies. AI can develop individualized dosing regimens by analyzing patient-specific factors such as body weight, organ function, and drug interactions. This personalized dosing ensures that patients receive the optimal amount of medication for their condition, balancing effectiveness with the risk of side effects. AI can recommend effective combination therapies by analyzing data on how different drugs interact and the outcomes of various treatment combinations. For example, in treating multiple myeloma, AI can identify the most effective combinations of drugs based on patient-specific genetic and clinical data^[8]^.

AI-powered CDSS provide clinicians with evidence-based recommendations and highlight relevant diagnostic and treatment options. These systems analyze patient data against extensive medical knowledge bases, helping clinicians make informed decisions about the most appropriate treatments for hematological disorders. AI can serve as a second opinion tool for clinicians by providing additional diagnostic insights and confirming treatment decisions. This second-opinion capability ensures that the chosen treatment plan is optimal and aligns with current best practices in hematology. AI can develop algorithmic guidelines for treatment based on large-scale data analysis and clinical research. These guidelines help clinicians follow best practices and make evidence-based decisions for individual patients, leading to more effective and personalized treatment plans. AI supports collaborative platforms where multidisciplinary teams can review patient data and treatment options. These platforms facilitate expert consultations and collaborative decision-making, ensuring that treatment plans are comprehensive and tailored to patient needs^[25]^.

Personalized treatment plans driven by AI result in more effective therapies, leading to better clinical outcomes for patients. By tailoring treatments to individual patient profiles, AI helps achieve higher remission rates, improved disease management, and enhanced quality of life^[21]^. AI’s ability to match treatments to individual genetic and clinical profiles reduces the risk of adverse effects and improves patient safety. Personalized therapies ensure that patients receive the most effective treatments with the least risk of side effects. Effective and personalized treatments reduce the need for additional interventions, hospitalizations, and prolonged treatments, resulting in cost savings for both patients and healthcare systems. AI-driven personalized care ensures that resources are used efficiently to achieve the best possible outcomes. Personalized treatment plans contribute to better long-term health management by addressing the specific needs of each patient and providing targeted follow-up care. This approach reduces the likelihood of disease recurrence and improves overall patient health^[26]^.

## Enhanced data analysis capabilities

AI has revolutionized data analysis in hematology, offering enhanced capabilities that improve diagnostic accuracy, streamline workflows, and support personalized treatment^[22]^. AI technologies, particularly those using machine learning and deep learning, can process and analyze vast amounts of hematological data rapidly and efficiently. Modern computational resources, such as high-performance GPUs and cloud-based platforms, enable the processing of large datasets generated from blood tests, genomic sequencing, and clinical records. For example, deep learning models can analyze thousands of blood smear images or millions of genomic variants to identify patterns relevant to diagnosing blood disorders like leukemia or anemia. AI systems can manage large-scale data through advanced data storage solutions and efficient retrieval methods. Data lakes and distributed databases are used to store diverse types of hematological data, including imaging, genetic, and clinical information. AI algorithms facilitate efficient data retrieval and integration, enabling clinicians to access and analyze comprehensive patient data quickly. For example, AI can integrate data from EHRs with genomic data to provide a holistic view of a patient’s condition. AI tools can automatically clean and validate data, correcting errors and standardizing formats to ensure high-quality datasets for analysis. Techniques such as anomaly detection and data normalization are used to identify and rectify inconsistencies in data from different sources. For instance, AI algorithms can detect and correct errors in blood test results or identify inconsistencies in patient records, improving the reliability of diagnostic information. AI systems employ advanced data preprocessing techniques to prepare raw data for analysis. This includes feature extraction, dimensionality reduction, and data transformation. For example, in genomic data analysis, AI can preprocess raw sequence data to identify relevant genetic variants and reduce data complexity for more accurate downstream analysis^[27]^.

AI utilizes supervised learning algorithms, such as support vector machines (SVMs) and decision trees, to detect patterns in labeled hematological data^[23]^. These algorithms are trained on historical data to recognize specific disease characteristics and classify new patient samples. For example, supervised learning can differentiate between malignant and benign blood cell abnormalities in leukemia diagnoses based on labeled training data. Unsupervised learning techniques, such as clustering and dimensionality reduction, are employed to discover patterns and relationships in unlabeled data. Algorithms like k-means clustering and principal component analysis (PCA) are used to identify subtypes of hematological disorders and uncover hidden structures in data. For example, unsupervised learning can reveal new subtypes of anemia or identify novel biomarkers for disease prediction. CNNs are deep learning models that excel at analyzing complex and high-dimensional data, such as medical images. In hematology, CNNs are used to analyze blood smear images, detect abnormalities, and classify different types of blood cells with high accuracy. For instance, CNNs can distinguish between normal and abnormal blood cell morphology, aiding in the diagnosis of conditions like sickle cell disease or leukemia. Recurrent Neural Networks (RNNs) and Long Short-Term Memory (LSTM) Networks are used for analyzing time-series data and sequential information. In hematology, these models can track changes in patient data over time to predict disease progression or treatment responses. For example, LSTM networks can analyze longitudinal patient data to predict the progression of chronic blood disorders and guide treatment decisions. AI integrates data from multiple omics layers, such as genomics, transcriptomics, proteomics, and metabolomics, to provide a comprehensive understanding of hematological disorders. This multi-omics approach enables the identification of complex disease mechanisms and the discovery of new therapeutic targets. For example, integrating genomic data with proteomic and metabolomic data can reveal new insights into the molecular pathways involved in leukemia. AI tools can synthesize data from various platforms and sources, including laboratory tests, imaging studies, and patient surveys. This cross-platform synthesis allows for a more complete assessment of a patient’s condition and facilitates the development of integrated diagnostic and treatment strategies. For example, combining imaging data with genetic information can improve the accuracy of cancer staging and treatment planning^[28]^.

AI can fuse data from EHRs to create comprehensive patient profiles that include clinical history, lab results, imaging studies, and treatment outcomes^[24]^. This data fusion enables a more thorough analysis of patient conditions and supports personalized treatment plans. For example, AI can integrate historical lab results, imaging studies, and patient symptoms to diagnose complex hematological conditions and recommend targeted therapies. AI systems can integrate real-time data from various sources, such as patient monitoring devices and remote health apps. This real-time integration allows for up-to-date analysis of patient conditions and timely adjustments to treatment plans. For example, integrating data from wearable devices with EHRs can provide real-time insights into patient health and facilitate proactive disease management. AI models assess the risk of developing hematological disorders and predict disease outcomes based on patient data. Techniques such as logistic regression and ensemble methods are used to create risk assessment models that help in early diagnosis and treatment planning. For example, predictive models can estimate the likelihood of progression from pre-leukemia conditions to full-blown leukemia, guiding early intervention strategies. AI employs survival analysis techniques, such as Cox proportional hazards models and Kaplan-Meier estimators, to predict patient survival rates and treatment outcomes. These models analyze patient data to estimate the probability of disease progression or remission over time. For instance, survival analysis can predict the outcomes of different treatment regimens for patients with AML^[29]^.

AI can simulate various treatment scenarios to evaluate potential outcomes and guide treatment decisions^[8]^. Simulation models use patient data to explore the effects of different therapeutic approaches and predict the results of treatment changes. For example, simulation models can assess the impact of different chemotherapy regimens on patient survival and response in hematological malignancies. AI tools support scenario-based planning by analyzing different treatment scenarios and their potential outcomes. This planning helps clinicians choose the most effective treatment strategies based on simulated data. For example, scenario analysis can explore the effects of combining different drugs or adjusting dosages for patients with complex blood disorders. AI-powered data visualization tools create interactive dashboards that display complex hematological data in an intuitive and accessible format^[26]^. These dashboards allow clinicians to explore data, identify trends, and make informed decisions. For example, interactive dashboards can present blood test results, imaging data, and patient history in a visual format that highlights key diagnostic features. AI enables advanced visual analytics techniques, such as heatmaps, scatter plots, and network graphs, to explore and interpret hematological data. These visual tools help clinicians understand data relationships, identify patterns, and communicate findings to patients and colleagues. For instance, heatmaps can visualize gene expression patterns in cancer research, revealing potential targets for therapy. AI can automate the generation of diagnostic reports, summarizing complex data into concise and actionable information. Automated reports include diagnostic findings, treatment recommendations, and follow-up plans, reducing the time spent on report writing and review. For example, AI-generated reports can present blood smear analysis results, highlighting abnormalities and suggesting further tests. AI tools offer customizable reporting options that allow clinicians to tailor reports based on specific needs and preferences. These tools assist in interpreting data and providing clear, actionable insights for treatment planning. For example, customized reports can focus on specific aspects of a patient’s condition, such as genetic mutations or treatment responses^[30]^.

## Support for clinical decision-making

AI has become a crucial tool in supporting clinical decision-making in hematology by providing advanced diagnostic insights, evidence-based recommendations, and decision support systems. AI’s contributions in this area enhance the accuracy of diagnoses, optimize treatment strategies, and improve patient outcomes. AI-powered CDSS offer evidence-based recommendations by analyzing patient data against vast medical knowledge bases^[25]^. These systems integrate data from EHRs, clinical guidelines, and research literature to provide clinicians with actionable insights. For example, a CDSS for AML might analyze patient symptoms, laboratory results, and genetic information to suggest potential diagnoses and treatment options based on the latest clinical guidelines and research. AI tools assist clinicians in diagnosing hematological disorders by analyzing complex datasets and identifying patterns that may not be immediately apparent. For instance, AI algorithms can analyze blood smear images to detect abnormalities in cell morphology, helping clinicians differentiate between types of anemia or leukemia. These tools provide diagnostic suggestions and highlight potential conditions based on comprehensive data analysis. AI algorithms use historical data and machine learning techniques to provide diagnostic assistance for various hematological conditions. For example, algorithms can evaluate patterns in blood cell counts and other lab results to propose possible diagnoses for conditions such as myelodysplastic syndromes (MDS) or chronic lymphocytic leukemia (CLL). These algorithms help clinicians make more accurate and timely diagnoses by providing a structured approach to data interpretation. AI-driven treatment decision algorithms evaluate patient data to recommend personalized treatment plans. These algorithms consider factors such as disease stage, genetic mutations, and previous treatment responses to suggest optimal therapies. For instance, algorithms for chronic myeloid leukemia (CML) can recommend treatment regimens based on the patient’s BCR-ABL mutation status and response to prior therapies^[31]^.

AI systems provide a second opinion by automatically reviewing diagnostic data and offering additional insights. For example, an AI tool might re-evaluate blood smear images or genetic test results to confirm or question an initial diagnosis. This automated review serves as a valuable backup for clinicians, helping to ensure the accuracy of diagnoses and reducing the risk of missed conditions. AI enables virtual expert consultations where clinicians can seek second opinions from AI-powered platforms or specialist networks. These platforms use AI to analyze patient data and generate diagnostic or treatment recommendations based on expert knowledge and consensus. This approach offers clinicians access to additional expertise and supports decision-making for complex or rare hematological disorders. Expert systems powered by AI provide specialized support for diagnosing and managing rare or complex hematological conditions. These systems use a knowledge base of clinical expertise and research findings to assist clinicians. For instance, an AI-based expert system for rare blood disorders might offer diagnostic suggestions and management strategies based on a comprehensive database of similar cases and outcomes. AI systems access and utilize extensive clinical knowledge repositories to support decision-making. These repositories contain data from clinical trials, case studies, and treatment guidelines, which AI analyzes to provide up-to-date information and recommendations. For example, AI tools can access repositories of clinical trial data to suggest new treatment options for patients with refractory hematological diseases^[32]^.

AI develops structured decision-making frameworks that guide clinicians through complex diagnostic and treatment processes. These models incorporate decision trees, flowcharts, and rule-based systems to help clinicians evaluate options and make informed decisions. For example, a decision-making framework for the treatment of lymphoma might include steps for assessing disease stage, selecting therapies, and planning follow-up care. AI systems use clinical practice guidelines to support decision-making. These systems integrate guidelines into the decision-making process, ensuring that recommendations are based on the most current and evidence-based practices. For instance, AI tools can use NCCN guidelines to recommend treatment plans for patients with hematological malignancies. AI supports collaborative decision-making through platforms that facilitate communication among multidisciplinary teams. These platforms enable the sharing of patient data, diagnostic findings, and treatment options among hematologists, oncologists, pathologists, and other specialists. For example, a collaborative platform for managing patients with multiple myeloma allows team members to discuss treatment strategies and review data collectively. AI platforms assist in decision-making for complex cases by providing tools for data integration, analysis, and scenario modeling. These tools help teams evaluate different treatment approaches and predict potential outcomes based on comprehensive patient data and clinical expertise^[8]^.

AI tools provide real-time diagnostic assistance by analyzing data as it is collected. For example, AI algorithms can process blood test results or imaging data in real-time, offering immediate feedback and diagnostic suggestions to clinicians. This real-time support accelerates the diagnostic process and facilitates timely treatment decisions. AI systems enable real-time monitoring of treatment effectiveness by analyzing patient data from ongoing therapies. For instance, AI tools can track changes in blood counts, disease markers, or patient-reported outcomes to assess the success of treatment and recommend adjustments. AI enables dynamic adjustments to treatment strategies based on real-time data. For example, AI algorithms can analyze patient responses to ongoing treatments and suggest modifications to drug dosages or therapy regimens. This adaptability ensures that treatments remain effective and responsive to patient needs. AI uses predictive analytics to forecast future patient conditions and treatment outcomes. These predictions inform decision-making by anticipating potential challenges and opportunities. For example, AI can predict the likelihood of disease recurrence and guide decisions about ongoing maintenance therapies. AI uses advanced visualization tools to present complex data in an accessible format. These tools include interactive dashboards, heatmaps, and 3D models that help clinicians interpret large datasets and identify key trends. For example, a heatmap might visualize gene expression data to highlight differences between normal and malignant cells. AI-driven graphical data analysis provides visual representations of patient data, such as flow cytometry plots or gene expression graphs. These visual aids help clinicians interpret diagnostic results and make informed treatment decisions. AI automates the generation of diagnostic and treatment reports, summarizing data findings and providing actionable insights. These reports include diagnostic summaries, treatment recommendations, and follow-up plans, helping clinicians communicate information effectively to patients and other healthcare providers. AI tools generate structured report formats that standardize the presentation of diagnostic and treatment information. These standardized formats ensure that reports are clear, consistent, and aligned with best practices in hematological care^[8,25,26]^.

AI provides simulation-based training tools for clinicians, offering realistic scenarios for practicing diagnostic and treatment skills^[27]^. These tools use virtual patients and simulated cases to help clinicians develop expertise in hematological diagnostics and decision-making. AI systems offer educational resources and real-time feedback to support clinician learning and development. For example, AI-driven platforms provide case studies, quizzes, and performance assessments to help clinicians improve their diagnostic and treatment skills. AI supports educational platforms that disseminate knowledge and best practices in hematology. These platforms offer access to the latest research findings, clinical guidelines, and expert opinions, helping clinicians stay updated on advancements in the field. AI facilitates collaborative learning opportunities through online forums, webinars, and virtual workshops. These opportunities allow clinicians to share knowledge, discuss challenges, and learn from experts in hematology. AI supports the ongoing development and refinement of decision support tools, ensuring that they evolve with advances in medical knowledge and technology. This continuous improvement leads to more accurate diagnoses and effective treatments over time. AI’s decision support capabilities offer scalable solutions that can be applied across various healthcare settings, improving diagnostic and treatment practices in diverse environments. AI drives transformative changes in hematology by advancing research efforts and developing innovative diagnostic and therapeutic approaches. These advancements contribute to a deeper understanding of hematological disorders and the creation of new treatment strategies. AI’s support for clinical decision-making leads to better patient outcomes and increased healthcare efficiency. Accurate diagnoses, personalized treatments, and efficient care processes improve patient health and reduce overall healthcare costs.

## Improved patient outcomes

AI has fundamentally transformed the field of hematology, leading to significant improvements in patient outcomes through enhanced diagnostic accuracy, personalized treatment strategies, and efficient care management^[28]^. AI-driven diagnostic algorithms significantly enhance the early detection of hematological disorders by analyzing complex datasets from blood tests, imaging studies, and genetic analyses. For example, CNNs and other machine learning models can identify subtle changes in blood smear images, enabling the early detection of conditions such as leukemia and lymphoma. Early diagnosis allows for timely interventions and improves the chances of successful treatment outcomes. AI technologies, such as deep learning-based image analysis and pattern recognition systems, provide tools for more accurate and reliable diagnoses. These tools analyze blood cell morphology, genetic mutations, and molecular biomarkers with high precision, reducing the likelihood of diagnostic errors. For instance, AI systems can identify rare genetic variants associated with conditions like MDS, leading to more precise diagnoses and targeted treatments. AI systems help reduce diagnostic errors by cross-referencing patient data with extensive medical knowledge bases and historical case data. For example, AI-powered CDSS can identify discrepancies between clinical findings and diagnostic criteria, alerting clinicians to potential errors. By minimizing diagnostic errors, AI improves patient outcomes and ensures that patients receive the correct diagnoses and appropriate treatments. AI technologies validate diagnostic findings through automated reviews and second opinions. Tools such as automated blood smear analysis and AI-based diagnostic aids provide clinicians with additional perspectives on test results, ensuring that diagnoses are accurate and reliable. This validation process reduces the risk of missed or incorrect diagnoses and supports better patient care.

AI enables the development of personalized treatment plans by analyzing patient-specific data, including genetic profiles, disease stage, and treatment history. For example, AI algorithms can evaluate the effectiveness of different treatment options for conditions such as acute myeloid leukemia (AML) based on individual patient data. Personalized treatment strategies ensure that patients receive therapies that are most likely to be effective for their specific condition, leading to better treatment outcomes^[25]^. AI supports precision medicine approaches by integrating multi-omics data, such as genomics, proteomics, and metabolomics, to design individualized treatment plans. These approaches use detailed patient data to select targeted therapies, optimize drug dosages, and predict treatment responses. For instance, AI-driven analysis of genetic mutations in CLL helps identify the most effective targeted therapies for individual patients. AI technologies optimize treatment regimens by analyzing patient data and predicting responses to various therapies. For example, AI algorithms can model the outcomes of different chemotherapy regimens for patients with ALL, helping clinicians choose the most effective treatment plan. Optimized treatment regimens improve patient outcomes by ensuring that patients receive therapies that maximize efficacy while minimizing side effects. AI supports adaptive treatment strategies by monitoring patient responses to therapies in real-time and adjusting treatment plans as needed. AI algorithms analyze ongoing data from blood tests, imaging studies, and patient reports to make dynamic adjustments to treatment regimens. This adaptability ensures that treatments remain effective throughout the course of care and improves patient outcomes.

AI technologies streamline healthcare processes by automating administrative tasks, such as appointment scheduling, test result management, and patient follow-up^[29]^. For example, AI-powered systems can automate the scheduling of lab tests and the management of patient appointments, reducing administrative burdens on healthcare providers and improving the efficiency of care delivery. AI enhances workflow efficiency by integrating data from various sources and providing tools for managing patient care. For instance, AI-driven platforms can centralize patient information, streamline communication between healthcare providers, and facilitate the coordination of care activities. Improved workflow efficiency leads to better patient management and more effective care. AI technologies enable real-time monitoring of patient health by analyzing data from wearable devices, electronic health records, and other sources. For example, AI systems can track vital signs, blood counts, and treatment responses to identify potential issues early and provide timely interventions. Real-time monitoring supports proactive care and improves patient outcomes by addressing issues before they escalate. AI supports virtual care and remote support for patients through telemedicine platforms and remote monitoring tools. These technologies enable patients to receive consultations, follow-up care, and treatment adjustments from the comfort of their homes. Virtual care improves access to healthcare services, especially for patients in remote or underserved areas, and enhances patient outcomes by providing ongoing support and monitoring.

AI contributes to the development of evidence-based clinical guidelines by analyzing large datasets of clinical trial results, research studies, and patient outcomes^[30]^. For example, AI tools can review data from multiple sources to generate updated treatment guidelines for conditions such as myeloma or sickle cell disease. Evidence-based guidelines ensure that clinical practices are aligned with the latest research and best practices. AI technologies facilitate the implementation and adherence to clinical guidelines by providing clinicians with access to up-to-date recommendations and best practices. For example, AI-powered decision support systems can integrate clinical guidelines into the decision-making process, helping clinicians adhere to evidence-based practices and improve patient outcomes. AI supports the translation of scientific knowledge into clinical practice by disseminating research findings, treatment protocols, and best practices to healthcare professionals. For example, AI-driven platforms can provide updates on recent research developments and clinical advancements in hematology. Effective knowledge translation improves patient care by ensuring that clinicians have access to the latest information. AI technologies offer educational resources for clinicians, including training modules, case studies, and interactive learning tools. These resources help clinicians stay informed about new developments in hematology and enhance their diagnostic and treatment skills. Ongoing education supports better patient outcomes by ensuring that clinicians are up-to-date with the latest advancements in the field.

AI technologies continue to advance, offering new tools and methods for improving patient care in hematology^[31]^. These advancements lead to sustained improvements in diagnostic accuracy, treatment effectiveness, and patient outcomes. For example, ongoing research into AI algorithms for hematological conditions may lead to the development of new diagnostic tools and treatment approaches. The long-term impact of AI on patient health includes improved survival rates, better management of chronic conditions, and enhanced quality of life for patients with hematological disorders. AI-driven innovations contribute to lasting improvements in patient care and support long-term health outcomes. AI-driven technologies provide scalable solutions that expand access to advanced care for patients with hematological disorders. These solutions make high-quality diagnostic and treatment options available to a broader range of patients, including those in underserved or remote areas. Increased access to advanced care improves patient outcomes and supports equitable healthcare delivery. AI’s global impact on hematological care includes the dissemination of advanced diagnostic and treatment technologies across different regions and healthcare systems. By sharing innovations and best practices, AI contributes to improvements in patient care worldwide and supports the advancement of hematology as a field.

## Challenges and limitations of AI in hematology

While AI holds immense promise in revolutionizing hematological disorder diagnosis, several challenges and limitations need consideration^[32]^. Access to high-quality, diverse datasets in hematology, especially for rare disorders or specific patient subgroups, may hinder AI model training and validation. Existing datasets may reflect biases in terms of demographics, leading to AI models that are not representative of diverse patient populations. Some AI models, particularly deep learning algorithms, operate as black boxes, making it challenging to interpret their decision-making processes. This lack of explainability raises concerns regarding trust and acceptance among clinicians. Clinician skepticism or reluctance in adopting AI-based tools due to unfamiliarity, perceived complexity, or skepticism regarding their clinical utility might hinder their widespread adoption. Difficulty in seamlessly integrating AI tools into existing clinical workflows and EHR systems might impede their effective utilization. AI applications may involve handling sensitive patient data, raising concerns about data privacy, informed consent, and compliance with data protection regulations (eg, HIPAA). Issues surrounding the liability and responsibility of AI-generated recommendations in clinical decision-making pose legal and ethical dilemmas^[29]^.

Ensuring the robustness and generalizability of AI models across different patient populations, clinical settings, and geographic regions remains a challenge^[33]^. AI model performance may vary based on the quality and diversity of input data, potentially leading to varying diagnostic accuracy in different scenarios. Training and deploying sophisticated AI models require substantial computational resources, which might be inaccessible or costly for some healthcare facilities. The need for specialized skills and expertise in AI development, deployment, and maintenance poses a challenge, especially in smaller healthcare settings with limited resources. AI models often rely on expert annotations or interpretations as the gold standard for training, which may be prone to human errors or subjectivity. Addressing these challenges necessitates concerted efforts in improving data quality, enhancing interpretability, fostering clinician trust, ensuring regulatory compliance, and integrating AI seamlessly into clinical workflows. Ethical considerations, robust validation methodologies, and ongoing research collaborations are essential to overcome these limitations and leverage the full potential of AI in hematology.^[34-36]^

## Future directions and innovations

The future of AI in hematology holds significant promise, with several potential directions and innovations poised to shape the field. Further refinement of AI algorithms for analyzing blood smears, bone marrow aspirates, and histopathological slides to achieve higher accuracy in identifying subtle abnormalities^[34]^. Integration of various diagnostic modalities (genomic, proteomic, and imaging data) into unified AI-driven diagnostic platforms for a more comprehensive assessment. Continued advancements in AI-guided genomic analysis to identify rare mutations and biomarkers, facilitating personalized treatment strategies tailored to individual patient profiles. Improved AI models predicting patient-specific responses to treatments, optimizing therapeutic regimens and minimizing adverse effects. Focus on developing AI models with enhanced interpretability and explainability, enabling clinicians to understand and trust AI-generated recommendations. Development of AI systems providing justifications or reasoning behind diagnostic or treatment suggestions for better clinical acceptance. Integration of AI tools into telehematology platforms for remote consultations, enabling efficient diagnosis and management of hematological disorders in underserved areas. Utilization of wearable devices and remote monitoring tools, coupled with AI analytics, for continuous tracking of hematological parameters and disease progression. Encouraging international collaborations and data sharing initiatives to build larger, more diverse datasets, fostering AI research in hematological disorders. Implementation of federated learning approaches allowing AI models to learn from decentralized datasets while ensuring data privacy and security. Development of robust ethical frameworks and guidelines addressing AI use in hematological disorder diagnosis, emphasizing patient privacy, consent, and responsible AI deployment. Formulation of clear regulatory policies governing AI applications in healthcare to ensure compliance, patient safety, and accountability. AI-driven predictive models assisting in identifying potential therapeutic targets, accelerating drug discovery and development for hematological disorders. Incorporating AI education and training into medical curricula to equip healthcare professionals with the skills needed to utilize AI tools effectively in clinical practice. These future directions and innovations underscore AI’s potential in advancing hematological disorder diagnosis, paving the way for more accurate, personalized, and efficient patient care. Embracing collaborative research, addressing ethical considerations, and continually refining AI technologies will be key in realizing these transformative advancements in hematology.

Table 1 shows summary of AI Applications in Hematological Disorder Diagnosis (provided by the authors).

**Table 1:** Summary of AI applications in hematological disorder diagnosis

Application	Description	Key benefits	Challenges
Automated Blood Smear Analysis	AI algorithms analyze peripheral blood smears to detect abnormalities.	High precision Reduces manual workload Quick results	Dependence on quality of smear images Limited generalization for rare disorders
Disease Prediction Models	ML models predict the risk of developing hematological disorders.	Early intervention Personalized treatment planning	Risk of overfitting to training data Requires diverse datasets
Genomic and Proteomic Analysis	AI interprets genomic/proteomic data to identify disease-associated mutations.	Facilitates targeted therapies Enables precision medicine	Complex data integration High computational requirements
Coagulation Disorder Evaluation	AI evaluates coagulation profiles to predict bleeding or thrombotic risks.	Rapid assessment Supports management of conditions like hemophilia and DIC	Challenges in handling incomplete or noisy data Limited application in complex coagulopathies
Integration with POCT	AI in point-of-care devices for quick diagnostic results.	Enhances accessibility in remote areas Real-time diagnosis	Device calibration issues Limited by battery life and hardware constraints
Explainable AI	AI models with transparent decision-making processes.	Improves clinician trust Facilitates clinical integration	Development of interpretable models is resource-intensive
Real-Time Decision Support	Integration with EHR systems for actionable insights.	Improves workflow efficiency Ensures continuous monitoring	Requires robust infrastructure Risk of alert fatigue for clinicians

## Conclusion

AI has emerged as a transformative force in the field of hematology, revolutionizing the diagnosis, treatment, and management of hematological disorders. AI’s role in hematology is multifaceted, encompassing advancements in diagnostic accuracy, personalized treatment strategies, and efficient care management. Through sophisticated machine learning algorithms and data analysis techniques, AI enhances the ability to detect hematological disorders at earlier stages, thus enabling timely and accurate diagnoses. AI-driven diagnostic tools, such as automated blood smear analysis and predictive modeling, have proven effective in identifying subtle disease markers and reducing diagnostic errors. These advancements lead to earlier intervention and more precise treatment options, ultimately improving patient outcomes.

Personalized medicine, powered by AI, represents another significant leap forward in hematological care. By leveraging patient-specific data, including genetic profiles and treatment histories, AI algorithms facilitate the development of tailored treatment plans that maximize therapeutic effectiveness and minimize adverse effects. This approach ensures that treatment regimens are customized to the individual needs of each patient, leading to better disease management and enhanced quality of life. Furthermore, AI-driven tools support adaptive treatment strategies, allowing for real-time adjustments based on patient responses, which improves the overall effectiveness of therapies. The efficiency and cost-effectiveness of AI technologies also play a crucial role in transforming hematological care. AI streamlines healthcare processes, from automating administrative tasks to optimizing treatment workflows, which results in more efficient care delivery and reduced healthcare costs. Additionally, AI-based decision support systems enhance clinical decision-making by integrating clinical guidelines, providing evidence-based recommendations, and offering virtual second opinions. These systems support clinicians in making informed decisions and improving patient care.

## Data Availability

Not applicable as this a review.

## Research registration unique identifying number (UIN)

Not applicable as this a review.
